# Clinical Characteristics and Outcomes of Hospitalized COVID-19 Patients with Different Variants of SARS-CoV-2 in a Tertiary Care Hospital, Thailand

**DOI:** 10.3390/tropicalmed9110266

**Published:** 2024-11-05

**Authors:** Kitchawan Hengkrawit, Juthamas Thananon, Kritakarn Telapol, Chirawat Chiewchalermsri

**Affiliations:** 1 Department of Pediatrics, Panyananthaphikkhu Chonprathan Medical Center, Srinakharinwirot University, Nonthaburi 11120, Thailand; kitchawan@g.swu.ac.th; 2 Department of Radiology, Panyananthaphikkhu Chonprathan Medical Center, Srinakharinwirot University, Nonthaburi 11120, Thailand; juthamast@g.swu.ac.th; 3 Department of Obstetrics and Gynecology, Panyananthaphikkhu Chonprathan Medical Center, Srinakharinwirot University, Nonthaburi 11120, Thailand; 4 Department of Medicine, Panyananthaphikkhu Chonprathan Medical Center, Srinakharinwirot University, Nonthaburi 11120, Thailand

**Keywords:** COVID-19, clinical, characteristic, Delta variant, Alpha variant, Omicron variant, treatment outcome

## Abstract

The different strains of SARS-CoV-2 were detected and labeled in 2021. Each strain differs in both clinical symptoms and severity. Previous studies found different clinical symptoms and treatment outcomes between outbreak waves; however, data in Southeast Asia were limited. This study collected data of hospitalized COVID-19 patients from a Tertiary hospital in Thailand between January 2020 and December 2023 and analyzed patients’ data in each outbreak wave using Pearson’s chi-square. A total of 1084 inpatients were included for analysis. The median age was 64 (IQR, 0.4–100) years. The patients were hospitalized in predominantly Alpha (22.78%), Delta (21.68%), and Omicron (5.07%) periods of the virus outbreak. The largest age group was elderly (over 65 years old) in all three variant of concern (VOC) periods; 82.65% of the patients had comorbidities, including 58.5% hypertension, 46.5% dyslipidemia, and 42.0% diabetes mellitus (DM). The study found pneumonia at 67.53%, septic shock at 4.61%, acute respiratory distress syndrome (ARDS) at 2.86%, and congestive heart failure at 0.83% in all age groups with no significant difference between outbreak periods. The overall mortality rate was 16.14%. A total of 75% of deaths occurred in patients over 65 years old. The mortality rates in each VOC period were 20.0% Delta, 19.83% Alpha, and 13.23% Omicron. In the elderly group, the mortality rates were Delta 15.32%, Alpha 11.75%, and Omicron 10.88%. The Omicron VOC was less severe than other variants, particularly in the elderly (≥65 years). There were no significant differences in the younger (<65 years) age group. The elderly still had more severe symptoms and the highest mortality rates in every wave of outbreak.

## 1. Introduction

COVID-19 is an emerging disease caused by severe acute respiratory syndrome coronavirus 2 (SARS-CoV-2) [1]. A systematic review and meta-analysis of 17,515 patients revealed the clinical presentation of COVID-19 to be fever, cough, headache, fatigue, breathing difficulties, loss of smell, and loss of taste [2,3,4]. Most patients (81%) develop mild to moderate symptoms (including mild pneumonia). Critical symptoms (5%) include respiratory failure, shock, or multiorgan dysfunction [5,6]. Factors affecting symptom severity include patient characteristics (race, age, immunity status) and the particular strain of COVID-19 virus [2,3,4,5].

The virus strains of SARS-CoV-2 detected in 2021 were labeled by the WHO as Alpha, Beta, Gamma, Delta, and Omicron variants [6]. The WHO coordinated the Global Influenza Surveillance and Response System (GISRS) to monitor the COVID-19 variants for data regarding transmissibility, severity, postvaccination antibodies, and effectiveness of treatment modalities [7,8].

As the SARS-CoV-2 genome evolved into variants of concern (VOCs), the WHO reported outbreaks in waves [8,9,10]. In Thailand, the first wave occurred from the beginning of 2020 to the end of the year. The second wave, in which the B.1.36.16 variant was predominant, occurred from December 2020 to January 2021. In the third wave (April–June 2021), Alpha (B.1.1.7) was predominant. In the fourth wave (July–December 2021), Delta (B.1.617.2) was predominant, and in the fifth wave (January–December 2023), Omicron (B.1.1.529) was predominant [11].

The previous studies have compared clinical manifestations to treatment outcomes between various virus strains and found differences. Several studies and reviews show that overall, the Omicron VOC seems to be more transmissible than the other variants and was observed to cause less severe disease as well as have a lower risk of mortality including hospitalization, oxygen requirements, mechanical ventilation, and death [12,13,14,15,16,17,18,19]. However, there were some reviews that came to the opposite conclusion about mortality rate and among hospitalized patients [20,21,22]. However, the data from Southeast Asia was insufficient. Therefore, this work studied clinical differences between symptoms, severity, and treatment in each wave of outbreak in hospitalized patients in Thailand.

## 2. Materials and Methods

### 2.1. Methods

**Figure 1:**
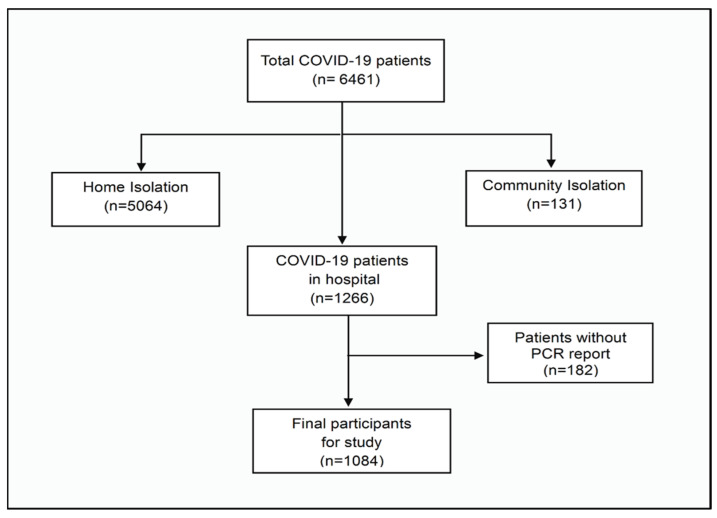
The participant flow chart represents excluded patients.

We collected data from the medical records of COVID-19 hospitalized patients at Panyananthaphikkhu Chonprathan Medical Center, Srinakharinwirot University (PCMC), Thailand between January 2020 and December 2023. All patients were confirmed to be diagnosed with COVID-19 by polymerase chain reaction (PCR). COVID-19 patients receive care including the decision to admit them as inpatients from doctors according to the treatment guidelines of the Thai Ministry of Public Health [23]. This has an indication for hospital treatment in cases where the patient has moderate to severe symptoms or mild symptoms but are at high-risk. This study reviewed demographic data, gender, comorbidity, clinical presentation, laboratory (first blood sampling after admission), complications, and treatment outcome including type of oxygen support. The severity of disease was defined by mild symptoms, where patents had an upper respiratory tract without pneumonia and no risk factors for severe disease; moderate was defined by patients with a respiratory tract illness with mild pneumonia (oxygen saturation ≥ 95%) with or without risk factors; severe was defined by pneumonia with hypoxia (oxygen saturation < 95%). The risk factors include elderly > 60 years, chronic lung, chronic kidney, heart disease or vascular disease, neuro disease, cancer, diabetic, obesity; BMI (body mass index) ≥ 30 kg/sqm, liver disease (Child–Pugh class B and above), immunocompromised, and HIV disease (CD4 cell count < 200 cells/cubic mm). The study excluded patients who had not had PCR reported (only rapid test result), and patients who were treated with home isolation and/or community isolation (patients showing few symptoms or a symptom) (Figure 1).

### 2.2. Epidermic Wave

Data analysis was divided into four-time frames: Pre-Alpha, Alpha, Delta, and Omicron, according to which VOC was dominant based on the genomic surveillance recorded by WHO/GISRS as over 70% of SARS-CoV-2 in Thailand [11]. Pre-Alpha (B.1.36.16) was predominant from December 2020 to January 2021. Alpha (B.1.1.7) was predominant from April to June 2021. Delta (B.1.617.2) was predominant from July to December 2021. Omicron (B.1.1.529) was predominant from January to December 2023.

### 2.3. Statistical Methods

The descriptive statistics and charts were done to describe demographic data, clinical manifestation, laboratory results, and the prevalence of SARS-CoV-2. The length of stay in the hospital was described as the mean and standard deviation (SD), while laboratory data were described as the median and interquartile range. All categorical variables were displayed as frequency (percentages). We compared categorical variables among variants of SARS-CoV-2 by using Pearson’s chi-square test or likelihood chi-square test. The differentiations in continuous variables with normality were assessed by analysis of variance (ANOVA) and a median test was used to analyzed continuous variables without normality. Statistical significance was identified using a two-sided *p*-value less than 0.05. IBM SPSS Statistics version 21 (IBM Corp, Armonk, NY, USA) was used as the management of data and analysis tool.

## 3. Results

### 3.1. Characteristics of Patients

**Figure 2:**
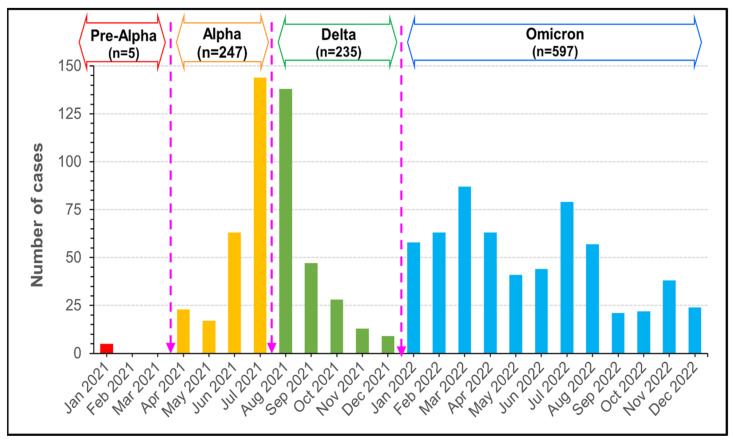
The number of monthly reported cases of COVID-19 during Pre-Alpha, Alpha, Alpha, Delta and Omicron variants in Thailand from January 2021 to December 2022.

A total of 1084 patients were included in the study, beginning in the second VOC outbreak period (Pre-Alpha). The majority of cases, 55.07% (597/1084), occurred during the Omicron phase. During the Alpha and Delta outbreaks there were 22.78% (247/1084) and 21.68% (235/1084) cases, respectively (Figure 2).

**Figure 3:**
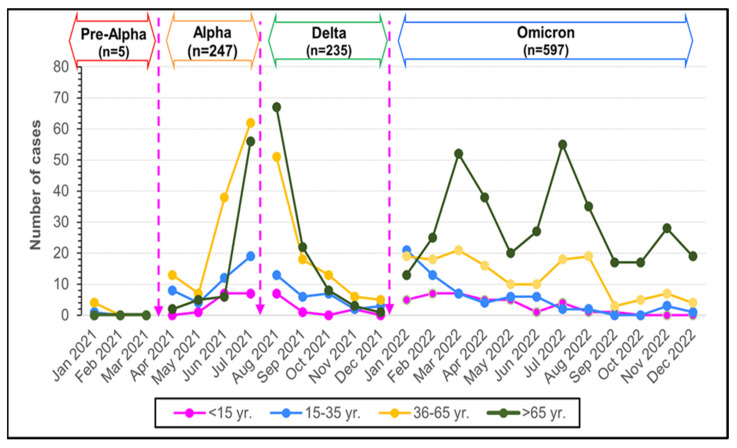
The number of monthly reported cases of COVID-19 during Pre-Alpha, Alpha, Delta, and Omicron variants in Thailand from January 2021 to December 2022 stratified by age groups (years).

The median age of the participants was 64 (IQR, 0.4–100) years. A total of 42.71% (463/1084) were male. The largest age group of patients was older than 65 years, accounting for 63.83% (682/1081), including 57.95% (346/682) of Omicron cases and 42.94% (101/682) of Delta cases. The middle age group (36–65 years) had the highest proportion of Alpha cases. Pediatric patients (≤15 years) were the smallest group, with a median age of 7 (IQR, 0.3–14) years, 59% (36/61) of which were ill during the Omicron outbreak (Figure 3).

A total of 82.65% of patients had comorbidities. The most common comorbidities were hypertension 58.5%, dyslipidemia 46.5%, and diabetes mellitus (DM) 42.0%. Hypertension 50%, dyslipidemia 64.8%, and heart disease 12.1% were mostly found in the Omicron phase significantly more than in other periods; (*p* = 0.001), (*p* = 0.001), (*p* = 0.001), respectively, while diabetes was found primarily in the Delta phase (*p* = 0.23) (Table 1).

Among patients younger than 65 years, there were 568 cases (52.39%). Among those, 42.5% of the Delta cases had comorbid hypertension and 31.3% had DM, *p* = 0.032, and *p* = 0.034, respectively. Heart disease was significant in the elderly group (*p* = 0.019) (Table 1) (Figure 2). There were twenty-six pregnant women; their median age was 26 (IQR, 18–42) years. A total of 76.92% (20/26) of the pregnant women were ill in the Omicron phase, two in the Alpha phase, and three in the Delta phase.

### 3.2. Clinical Manifestation

**Figure 4:**
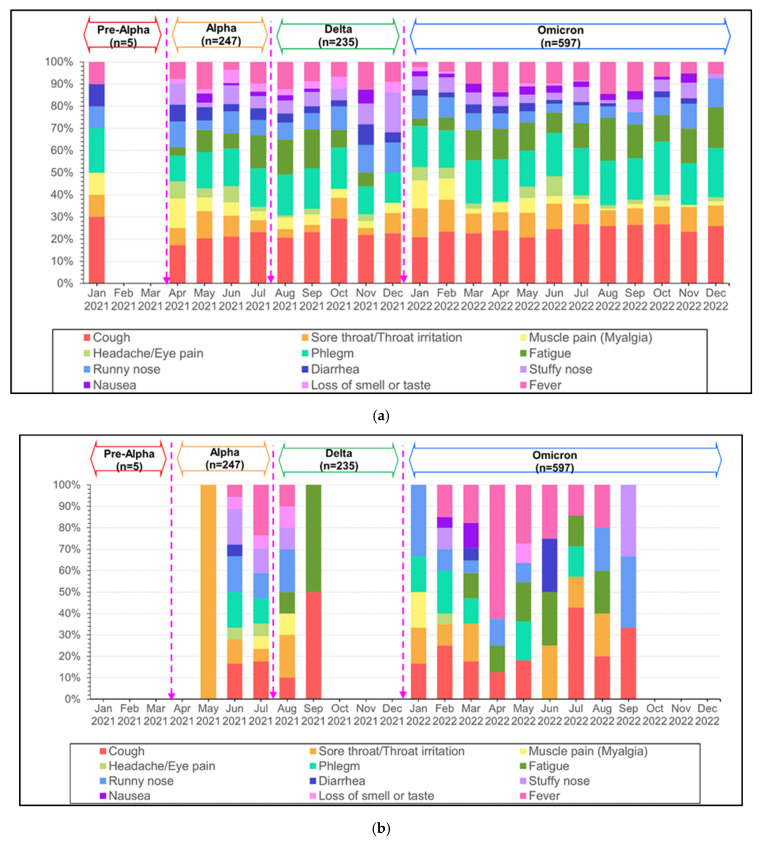

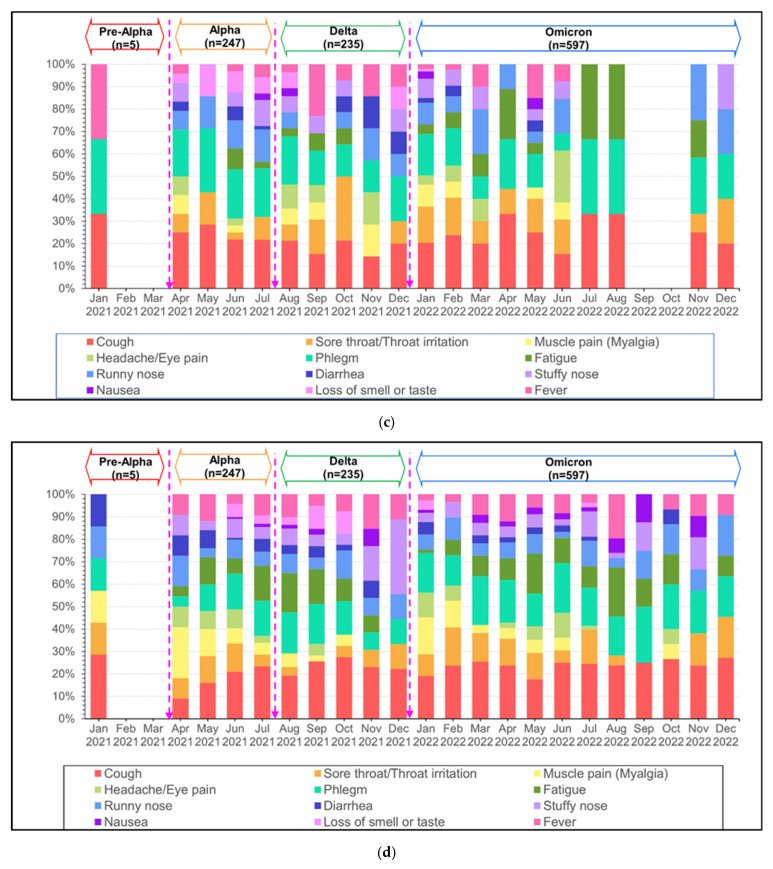

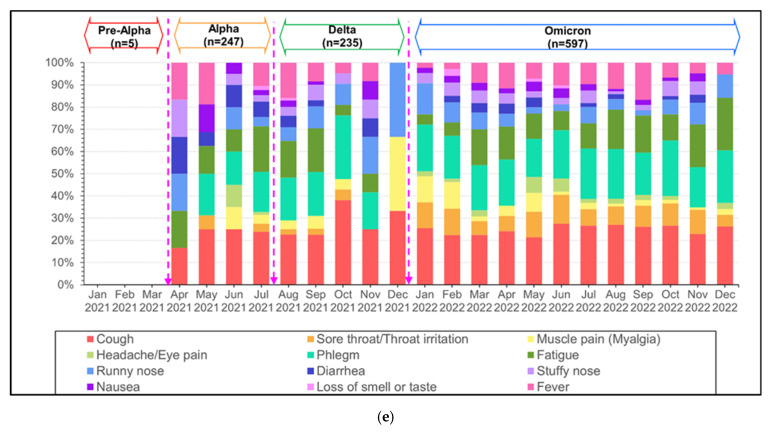
Symptoms from COVID-19 infections in Thailand from January 2021 to December 2022: (**a**) all patients; (**b**) age < 15 years; (**c**) age 15–35 years; (**d**) age 36–65 years; and (**e**) age > 65 years.

Overall, typical clinical manifestations were cough 66.2%, phlegm 52.9%, fatigue 34.6%, fever 28.6%, and sore throat. Headache and diarrhea were significantly more common in the Alpha phase (*p* = 0.007), (*p* = 0.003), and sore throat was more common in the Omicron phase (*p* = 0.001). Cough, phlegm, and fatigue were the top three most common symptoms during every outbreak (Table 2) (Figure 4).

In the under 65-year-old group, the top three clinical manifestations were cough 63.4%, phlegm 48.9%, and fatigue 28.2%. Sore throat was common during the Omicron outbreak, *p* < 0.001. Loss of smell or taste were common symptoms during the Alpha VOC (*p* < 0.001). Thirteen pediatric patients (≤15 years) were found to have symptoms of croup, with the highest proportion occurring in the Omicron period, 24.39% (10/41) (Table 2) (Figure 3). Croup symptoms were found in 22.22% (2/9) and 7.14% (1/14) (*p* = 0.012) of children in the Alpha and Delta periods, respectively. There were (21/64) 32.81% cases of seizure, including eighteen febrile convulsion and three fever provoked seizures. A total of 39% (16/41), 21.42% (3/14), and 22.22% (2/9) were in Omicron, Alpha, and Delta dominant periods (*p* = 0.051).

In the elderly group, fatigue and diarrhea were found to be more significant in the Alpha period than in other phases (*p* = 0.006) (*p* < 0.001). Sore throat and headache were significantly more prevalent in the Omicron period (*p* < 0.001) (*p* < 0.030) (Table 2) (Figure 4).

In the pregnant group, 69.23% (18/26) were asymptomatic. Cough was the most common symptom (53%), followed by runny nose, sore throat, phlegm, headache, debility, and muscle pain, at 48%, 44%, 28%, 25%, 20%, and 12%, respectively. Preterm labor occurred in the Omicron, Alpha, and Delta VOC periods, at 48.28%, 38%, and 33%, respectively. In the Omicron period, cough was common. Sore throat was prevalent in the Alpha and Delta periods. Preeclampsia with no severe respiratory symptoms occurred in 11.53% (3/26) of cases. Only 3.8% (1/26) of 5-minute Apgar scores were <7; however, after resuscitation, each baby returned to normal, and none of the children were infected with COVID-19.

### 3.3. Laboratory

Overall, in the Pre-Alpha period, median white blood cell count, 6000 cell/µL (IQR, 4–9) (*p* = 0.014), and neutrophil, 54% (IQR, 49–65) (*p* = 0.001), were significantly lower than at other periods. In the Delta period, lactate dehydrogenase level, 360 U/L (IQR, 268–496) (*p* < 0.001), was significantly higher than in other periods. In the Alpha period, median of c-reactive protein, 35 mg/L (IQR, 9–82) (*p* = 0.003), was significantly higher than in other periods. In elderly patients, lactate dehydrogenase level, 396 U/L (IQR, 280–511) (*p* < 0.001), was higher in the Delta period, and c-reactive protein, 48 mg/L (IQR, 9–84) (*p* = 0.001), was higher in the Alpha period. See Table 3.

### 3.4. Severity and Mortality

**Figure 5:**
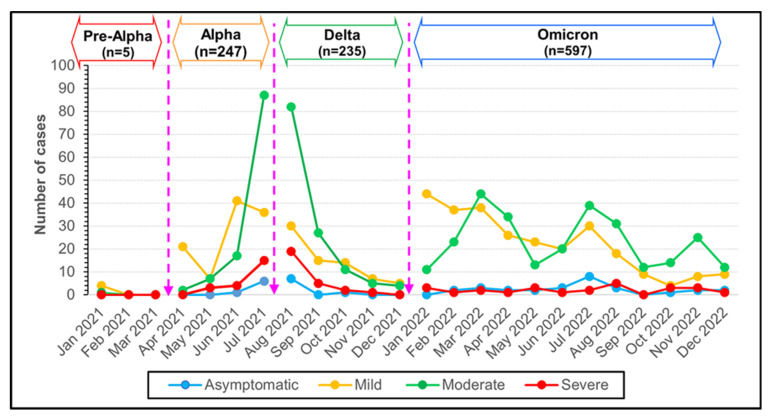
The number of monthly reported cases of COVID-19 during Pre-Alpha, Alpha, Delta and Omicron variants in Thailand from January 2021 to December 2022 stratified by COVID-19 severity classification.

The patients in the Alpha and Delta periods mostly had moderate symptoms, and approximately 80–90% occurred between July 2021 and September 2021, while severe symptoms occurred in 18% and 20% of cases. There were fewer moderate and severe symptoms in the Omicron period, with the peak values being 40% and 30% in March 2022 and August 2022, respectively (Figure 5).

**Figure 6:**
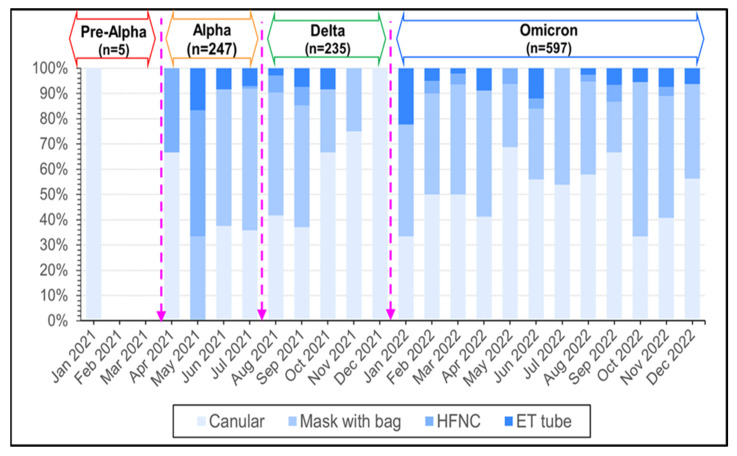
The number of monthly reported cases of COVID-19 during Pre-Alpha, Alpha, Alpha, Delta, and Omicron variants in Thailand from January 2021 to December 2022 stratified by oxygen respiratory support within 48 h.

In the group of patients who needed oxygen, there were 4.05%, 2.55%, and 2.68% patients on endotracheal (ET) tubes in the Alpha, Delta, and Omicron periods, respectively (Figure 5). However, there were no significant differences between outbreak periods regarding oxygen support equipment (Table 4) (Figure 6).

The mean of length of stay was 10.8 ± 9.3 days, and the median was 9 days (IQR; 6–12) for all outbreak periods. During the Alpha period, patients stayed in the hospital significantly longer in all age groups (Table 4). Among patients younger than 65 years, the median length of stay at the hospital was longest in the Pre-Alpha period and the mean length of stay was longest in the Delta period (Table 5).

In all age groups there were no significant differences between outbreaks in terms of pneumonia 67.53% (732/1084), septic shock 4.61% (50), acute respiratory distress syndrome (ARDS) 2.86% (31/1084), and congestive heart failure 0.83% (9/1084).

There were 175 deaths; the overall mortality rate was 16.14% [175/1084]. In the death group, 75% (130/175) were over 65 years of age and 25% (45/175) were under 65 years of age. The study showed similar mortality rates for Delta and Alpha at 20.0% (47/235) and 19.83% (49/247), while the Omicron period had the lowest mortality rate at 13.23% (79/597). For the elderly group, the mortality rate in the Delta period was higher than in the Alpha and Omicron periods (15.32% (36/235) vs. 11.75% (29/247) and 10.88% (65/597)). In the under 65-year-old group the mortality rate in the Alpha period was higher than in the Delta and Omicron periods, at 8.09% (20/247), 4.68% (11/235), and 2.34% (14/597), respectively.

**Figure 7:**
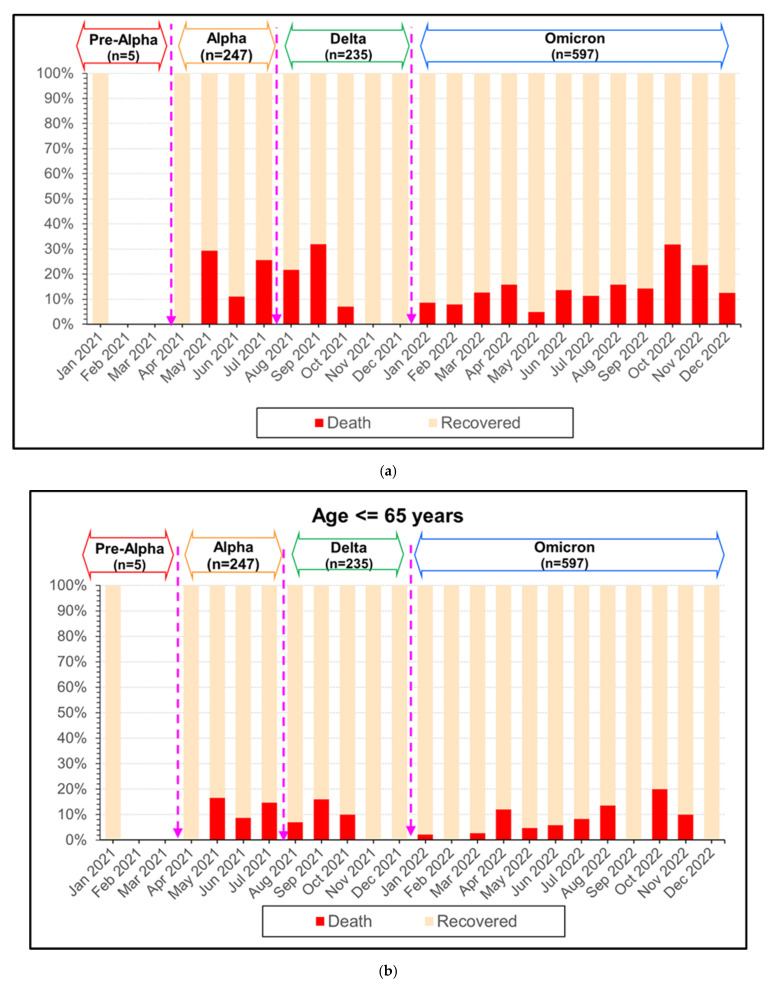

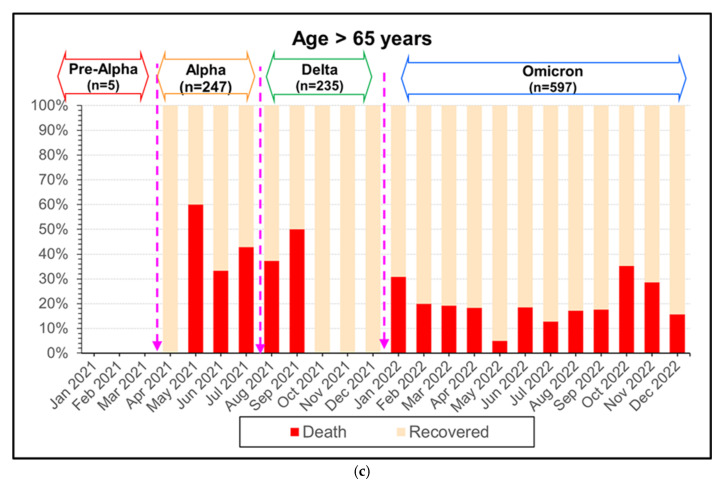
The number of monthly reported COVID-19 deaths during Pre-Alpha, Alpha, Delta, and Omicron variants in Thailand from January 2021 to December 2022; (**a**) all patients; (**b**) age ≤ 65 years; and (**c**) age > 65 years.

The proportions of mortality cases exhibited similarly high peaks in the Alpha, Delta, and Omicron periods (29%, 32%, and 32%). However, in the elderly group, the peak mortality in the Alpha period was higher than in the Delta and Omicron periods (60% vs. 50% vs. 35%) (Figure 7).

### 3.5. Highlights of Each Outbreak Period

#### 3.5.1. The Alpha Outbreak

The Alpha VOC was the first big outbreak in Thailand. The patients in this period, the middle age group (36–65 years), predominated (21.68%) (Figure 2). The patients in this VOC had three common comorbidities: hypertension, DM, and hyperlipidemia, similar to other VOCs. The study still found significantly more patients with cirrhosis (1.6%, *p* = 0.007) than in any other period (Table 1), except for three (cough, phlegm, fatigue) common clinical manifestations that had the same outbreak period. The study also found that fatigue, headache, diarrhea, and loss of smell or taste (14.6%) were significantly more than in other periods (*p* = 0.006), (*p* = 0.007), (*p* = 0.003), (*p* < 0.001) (Table 2). Laboratory patients in Alpha showed significantly higher levels of lymphocyte (25%) and c-reactive protein (25 mg/L) than in other VOCs (*p* < 0.001), (*p* < 0.003) (Table 3).

#### 3.5.2. The Delta Outbreak

In this outbreak period, the elderly age group was the highest. Cough, phlegm, and fatigue were the top three most common symptoms similar to the others. However, sore throat (12.3%, *p* < 0.001) and headache/eye pain (3.4%, *p* = 0.007) were found less frequently than at other VOC. The median of lactate dehydrogenase in the Delta VOC was higher than the other groups in both all ages and elderly groups (360 U/L) (*p* < 0.001) (Table 3). The study also showed that the severity of clinical (20%), mortality rates (20.0%), and mean of length of stay (16.6 ± 44.5) in this VOC is highest when compared to other periods (Table 5) (Figure 5).

#### 3.5.3. The Omicron Outbreak

This period had the most patients (55.07%), and the elderly were common. The patients with comorbid disease were found to have more heart disease than the other patients. For the patients in this VOC, sore throat (12.3%, *p* = 0.001) was predominate. This work found more pediatric patients than in other periods (59%) and also found croup (24.39%) and febrile seizures (32.81%) in children more than in other periods. This study shows that COVID-infected mothers gave birth to the highest number of premature babies. (48.28%) com-pared to other periods and the disease severity in Omicron is less than in Alpha and Delta; Including an overview of death, especially in the elderly.

## 4. Discussion

Previous studies suggest that the severity of SARS-CoV-2 outbreaks is decreasing, although symptoms, severity, and treatment results of each outbreak differ between nationalities and between public health systems [24,25].

Our research found more patients during the Omicron outbreak than during any other VOC outbreak, which was consistent with studies of Hu FH et al. [13] and Miyashita K et al. [14] in Japan. In contrast, a multicenter retrospective study from Iran [22] and another from Southern Africa [17] found that the number of patients during the Omicron VOC period was lower than in previous VOC outbreaks because both of those studies collected inpatient data; Omicron symptoms were milder, so fewer patients needed hospitalization.

The Beheshti Namdar A et al. [26] study demonstrated the nucleotide sequence for SARS-CoV-2 and determined that Omicron mutated by L452Q substitution, which increased ACE2-binding, leading to higher transmissibility than in other variants.

The elderly were the largest age group overall, 63.83%, and they were the largest age group in each wave of outbreak. Studies in China of epidemiological characteristics [13,20] and big data reviews [13,25] found that 75–87% of COVID-19 patients were elderly.

In our study and in studies by Siordia JA Jr. et al. [27] and Zizza A. et al. [28], hypertension was the most common comorbidity overall and in each VOC. The populations of these studies were mostly elderly, and the elderly are at higher risk of hypertension than any other age group. A large cohort study from Iran (24,287 hospitalized patients) found that heart disease was more significant than any other disease [24]. In our study, heart disease was common in the elderly group (*p* = 0.019) and it was related to high mortality, as it was in a study by Kimball A. et al. [29].

Previous studies of clinical presentations of patients with COVID-19 showed that the three main symptoms were fever 58.9–82.2%, cough 61.7–64.8%, and fatigue 27.2–44.0% [28,29,30,31]. This study found that respiratory symptoms were more common than fever symptoms, and there were fewer gastrointestinal symptoms in this study than in previous studies [28,29,30,31].

The results of this study indicate that the overall severity of illness, length of hospital stay, and proportion of mortality were higher during the Alpha phase than during the Delta or Omicron phases. Our mortality rates for Alpha, Delta, and Omicron were 20.0%, 19.83%, and 13.23%, respectively. These figures were close to those of Jassat W. et al. [32] (28.8%, 26.4%, and 10.7%) and Karuniawati A. et al. [30] (24.4%, 9.6%, and 11.9%), but higher than those of Shirafkan H. et al. [24] (6.8%, 2.7%, and 3.5%) and Sadeghi F. et al. [22] (6.8%, 2.7%, and 3.5%). Consistent with many previous studies [12,22,23,24,25], we found higher mortality rates among elderly patients than among younger patients.

Our study found that pneumonia, presenting in 67.53% of patients, was the most common complication. This proportion is higher than the 30.5–48.5% found in previous studies. In contrast, only 2.86% of patients in our study were diagnosed with ARDS, whereas previous studies reported 41.8–60.7% of patients with ARDS [20,21,23].

Previous virology studies reported that Omicron was more contagious than other variants but produced less severe symptoms [22,24,25]. Omicron is less severe because it affects primarily the upper respiratory tract and doesn’t spread deeper into the lungs [31]. A study from the US reported that 80% of deaths due to COVID-19 occurred in patients older than 65 years, while the highest proportion of severe outcomes was found among people over 85 years old.

The previous systemic review study of pregnant COVID-19 patients found 91.8% with pneumonia, 27.0% with dyspnea, 16.2% with preeclampsia, 64.7% with miscarriage, 41.1% with preterm labor, 11.7% with fetal growth restriction, and 57.2% of newborns admitted to a neonatal intensive care unit [31]. In our study, most pregnant patients were asymptomatic or had only mild symptoms. It is possible that the review study documented only the first wave of COVID-19 outbreaks, before effective vaccines were available. In our study, as in several previous studies, pregnant women who had received at least one vaccination exhibited milder symptoms than those who had not been vaccinated [17,32,33,34]. However, this work had no history of vaccination for analysis.

Like the systemic review studies [35], we found that pediatric patients had less severe symptoms than adult and elderly patients. Previous studies reported fever and cough in 47–70% and 30–49% of pediatric patients. Our findings were slightly lower. Previous studies reported gastrointestinal symptoms in 11–31% of pediatric patients, and less in adults. The proportion in adults was lower in our study, and there was no significant difference between VOC outbreaks. Other studies reported 0.8–8.7% loss of smell or taste, which was higher than our findings. It may be difficult for young children to identify these symptoms.

This study found more seizures than previous studies. All seizures occurred in children 1–3 years old, and they were most common in the Omicron period, which is consistent with many other Asian studies [2,3,4].

Many previous Asian studies found croup (24.39%) in the Omicron VOC period. Our findings were slightly higher (24.39%). All Asian studies found croup in patients younger than 5 years [1,2,3,4], while American and European studies found croup in children older than 6 years [5,6].

Consistent with previous studies [5,6], we found clinical symptoms, ICU admission rates, and mortality rates more severe in the Alpha and Delta periods than in the Omicron period.

This study found three cases of MIS-C in pediatric patients in the Alpha period. Laird-Gion J et al. [36] found one hundred and eight cases between 2020 and 2023, and 75% of the cases were in the Omicron period. This may be due to racial differences. The number of premature births was not significantly different between VOC outbreaks. No child contracted the virus from his/her mother.

For elderly patients, symptoms were less severe, and mortality rates were lower during the Omicron outbreak than during previous outbreaks. For other age groups, symptoms and mortality rates were not significantly different between outbreaks, least of all for children.

This study shows significantly higher lactate dehydrogenase levels in the Delta VOC and C-reactive proteins in the Alpha VOC, particularly in the elderly. Lactate dehydrogenase and C-reactive proteins are related to disease severity and mortality; however, further studies are needed to confirm any association between them. Other laboratory results, e.g., white blood cell count, neutrophil, and albumin level, differed significantly between VOCs but were still within normal limits.

From 12 January 2020 to 31 December 2023, Thailand reported a total of 4,715,152 confirmed COVID-19 patients. During this period, the cumulative number of new COVID-19 patients was approximately 4,292,491. The percentage of COVID-19 patients who required hospitalization was around 11.1% [37]. For this study, the hospitalized rate was 16.77%, which is slightly higher because the hospital is at a tertiary level.

Severity and mortality rates were affected by public health measures such as national lockdown, screening before entering the country, quarantine, and especially, vaccination policy. Thailand implemented vaccinations in February 2021 [38], during the Alpha VOC period. By then, Thailand had accumulated thirteen million doses, enough for at least one dose per person, including the 2.8 million people in high-risk groups. By the end of the Delta period, one hundred million doses had been distributed, including fifty-one million doses to the elderly, chronically diseased persons, and pregnant women. More than 70% of the population had been vaccinated, resulting in less severe symptoms during the Omicron outbreak.

Regulations issued by the Ministry of Public Health also had an impact on admission rates. During the Alpha VOC period, patients had to be quarantined for at least fourteen days despite being clinically fine and declined to ten and five days in Delta and Omicron VOCs. Although the coronavirus disease (COVID-19) appears to be less severe now, the measures for care appear to be relaxed. However, the COVID-19 disease has not disappeared and there are constant mutations that affect the clinical manifestations, which are difficult to predict. Therefore, continuous monitoring studies are necessary and the promotion of vaccinations by the Ministry of Public Health should be done continuously, particularly for high-risk group.

## 5. Conclusions

Hospitalized patients in the Omicron outbreak had less severe symptoms and a lower death rate than in the other outbreaks, particularly in the elderly. For middle-aged adults, children, and pregnant women, the effects did not differ significantly between variants. Risk of severe symptoms and death were significantly higher in patients over 65 years old. However, the SARS-CoV-2 virus continues to mutate. The specific clinical effects of the virus in different populations still need to be studied.

**Limitations:** The retrospective data was less detailed than we would have preferred. Particularly, vaccination status in this work had a record of only 10%, which cannot be analyzed. In addition, some young children or elderly patients may not have been able to provide information about certain symptoms, such as ability to smell or taste. For some groups of patients, such as pregnant women, infants, and children, the sample size may have been too small to draw conclusions and should be further studied. Furthermore, this study analyzed data on hospitalized patients only and did not include outpatients. The treatment guidelines of the Thai Ministry of Public Health were revised several times during the early stages of the outbreak, specifically regarding the type and dosage of antiretroviral drugs.

## Figures and Tables

**Table 1:** The comorbidities of COVID-19 patients by outbreak period.

Comorbidity	Overall, n (%)	SARS-CoV-2 Variant, n (%)				*p*-Value
		Pre-Alpha	Alpha	Delta	Omicron	
(i) All ages						
N	1084	5	247	235	597	
Hypertension	634 (58.5)	1 (20.0)	101 (40.9)	145 (61.7)	387 (64.8)	<0.001
DM Type 1 & Type 2	347 (32.0)	1 (20.0)	60 (24.3)	84 (35.7)	202 (33.8)	0.023
Dyslipidemia	504 (46.5)	1 (20.0)	87 (35.2)	117 (49.8)	299 (50.1)	<0.001
Heart Disease	96 (8.9)	0 (0.0)	7 (2.8)	17 (7.2)	72 (12.1)	<0.001
Immunocompromised	17 (1.6)	0 (0.0)	3 (1.2)	8 (3.4)	6 (1.0)	0.084
Cirrhosis	16 (1.5)	1 (20.0)	4 (1.6)	3 (1.3)	8 (1.3)	0.007
Asthma/Allergic Rhinitis	51 (4.7)	0 (0.0)	6 (2.4)	15 (6.4)	30 (5.0)	0.194
Others	600 (55.4)	1 (20.0)	105 (42.5)	115 (48.9)	379 (63.5)	<0.001
(ii) Age < 65 years						
N	568	5	178	134	251	
Hypertension	206 (36.3)	1 (20.0)	50 (28.1)	57 (42.5)	98 (39.0)	0.032
DM Type 1 & Type 2	129 (22.7)	1 (20.0)	31 (17.4)	42 (31.3)	55 (21.9)	0.034
Dyslipidemia	173 (30.5)	1 (20.0)	51 (28.7)	49 (36.6)	72 (28.7)	0.352
Heart Disease	20 (3.5)	0 (0.0)	3 (1.7)	6 (4.5)	11 (4.4)	0.414
Immunocompromised	16 (2.8)	0 (0.0)	3 (1.7)	8 (6.0)	5 (2.0)	0.091
Cirrhosis	11 (1.9)	1 (20.0)	3 (1.7)	2 (1.5)	5 (2.0)	0.032
Asthma/Allergic Rhinitis	27 (4.8)	0 (0.0)	5 (2.8)	8 (6.0)	14 (5.6)	0.466
Others	264 (46.5)	1 (20.0)	71 (39.9)	54 (40.3)	138 (55.0)	0.003
(iii) Age > 65 years						
N	516	0	69	101	346	
Hypertension	428 (82.9)	0 (0.0)	51 (73.9)	88 (87.1)	289 (83.5)	0.070
DM Type 1 & Type 2	218 (42.2)	0 (0.0)	29 (42.0)	42 (41.6)	147 (42.5)	0.986
Dyslipidemia	331 (64.1)	0 (0.0)	36 (52.2)	68 (67.3)	227 (65.6)	0.079
Heart Disease	76 (14.7)	0 (0.0)	4 (5.8)	11 (10.9)	61 (17.6)	0.019
Immunocompromised	1 (0.2)	0 (0.0)	0 (0.0)	0 (0.0)	1 (0.3)	0.782
Cirrhosis	5 (1.0)	0 (0.0)	1 (1.4)	1 (1.0)	3 (0.9)	0.903
Asthma/Allergic Rhinitis	24 (4.7)	0 (0.0)	1 (1.4)	7 (6.9)	16 (4.6)	0.249
Others	336 (65.1)	0 (0.0)	34 (49.3)	61 (60.4)	241 (69.7)	0.003

**Table 2:** Symptoms of COVID-19 patients by VOC period.

Symptom		Overall, n (%)	SARS-CoV-2 Variant, n (%)				*p*-Value
			Pre-Alpha (*n* = 5)	Alpha (*n* = 247)	Delta (*n* = 235)	Omicron (*n* = 597)	
(i) All ages							
N		1084	5	247	235	597	
Fever (>37 °C)		310 (28.6)	1 (20.0)	67 (27.1)	82 (34.9)	160 (26.8)	0.312
Cough		718 (66.2)	3 (60.0)	163 (66.0)	144 (61.3)	408 (68.3)	0.277
Sore throat		258 (23.8)	1 (20.0)	52 (21.1)	29 (12.3)	176 (29.5)	<0.001
Muscle pain (Myalgia)		151 (13.9)	1 (20.0)	39 (15.8)	31 (13.2)	80 (13.4)	0.774
Headache/Eye pain		93 (8.6)	0 (0.0)	29 (11.7)	8 (3.4)	56 (9.4)	0.007
Phlegm		573 (52.9)	2 (40.0)	124 (50.2)	116 (49.4)	331 (55.4)	0.286
Fatigue		375 (34.6)	0 (0.0)	87 (35.2)	92 (39.1)	196 (32.8)	0.129
Runny nose		246 (22.7)	1 (20.0)	58 (23.5)	54 (23.0)	133 (22.3)	0.981
Diarrhea		106 (9.8)	1 (20.0)	37 (15.0)	26 (11.1)	42 (7.0)	0.003
Stuffy nose		176 (16.2)	0 (0.0)	47 (19.0)	42 (17.9)	87 (14.6)	0.255
Nausea		73 (6.7)	0 (0.0)	13 (5.3)	13 (5.5)	47 (7.9)	0.394
Loss of smell or taste		59 (5.4)	0 (0.0)	29 (11.7)	20 (8.5)	10 (1.7)	<0.001
(ii) Age < 65 years							
N		568	5	178	134	251	
Fever (>37 °C)		155 (27.3)	1 (20.0)	43 (24.2)	41 (30.6)	70 (27.9)	0.763
Cough		360 (63.4)	3 (60.0)	112 (62.9)	76 (56.7)	169 (67.3)	0.232
Sore throat		158 (27.8)	1 (20.0)	45 (25.3)	22 (16.4)	90 (35.9)	<0.001
Muscle pain (Myalgia)		92 (16.2)	1 (20.0)	30 (16.9)	18 (13.4)	43 (17.1)	0.795
Headache/Eye pain		66 (11.6)	0 (0.0)	25 (14.0)	8 (6.0)	33 (13.1)	0.093
Phlegm		278 (48.9)	2 (40.0)	87 (48.9)	60 (44.8)	129 (51.4)	0.638
Fatigue		160 (28.2)	0 (0.0)	47 (26.4)	47 (35.1)	66 (26.3)	0.120
Runny nose		147 (25.9)	1 (20.0)	48 (27.0)	31 (23.1)	67 (26.7)	0.846
Diarrhea		54 (9.5)	1 (20.0)	21 (11.8)	14 (10.4)	18 (7.2)	0.327
Stuffy nose		112 (19.7)	0 (0.0)	40 (22.5)	28 (20.9)	44 (17.5)	0.398
Nausea		32 (5.6)	0 (0.0)	6 (3.4)	6 (4.5)	20 (8.0)	0.178
Loss of smell or taste		50 (8.8)	0 (0.0)	26 (14.6)	18 (13.4)	6 (2.4)	<0.001
(iii) Age > 65 years							
N	516		0	69	101	346	
Fever (>37 °C)	155 (30.0)		0 (0)	24 (34.8)	41 (40.6)	90 (26.0)	0.065
Cough	358 (69.4)		0 (0)	51 (73.9)	68 (67.3)	239 (69.1)	0.643
Sore throat	100 (19.4)		0 (0)	7 (10.1)	7 (6.9)	86 (24.9)	<0.001
Muscle pain (Myalgia)	59 (11.4)		0 (0)	9 (13.0)	13 (12.9)	37 (10.7)	0.752
Headache/Eye pain	27 (5.2)		0 (0)	4 (5.8)	0 (0.0)	23 (6.6)	0.030
Phlegm	295 (57.2)		0 (0)	37 (53.6)	56 (55.4)	202 (58.4)	0.710
Fatigue	215 (41.7)		0 (0)	40 (58.0)	45 (44.6)	130 (37.6)	0.006
Runny nose	99 (19.2)		0 (0)	10 (14.5)	23 (22.8)	66 (19.1)	0.402
Diarrhea	52 (10.1)		0 (0)	16 (23.2)	12 (11.9)	24 (6.9)	<0.001
Stuffy nose	64 (12.4)		0 (0)	7 (10.1)	14 (13.9)	43 (12.4)	0.770
Nausea	41 (7.9)		0 (0)	7 (10.1)	7 (6.9)	27 (7.8)	0.738
Loss of smell or taste	9 (1.7)		0 (0)	3 (4.3)	2 (2.0)	4 (1.2)	0.177

**Table 3:** The laboratory results of COVID-19 patients by outbreak time.

Laboratory Results	Overall	SARS-CoV-2 Variant				*p*-Value
		Pre-Alpha	Alpha	Delta	Omicron	
(i) All ages						
Median (IQR)						
Hemoglobin (g/dL)	12 (7–15)	11 (8–15)	12 (9–14)	12 (8–13)	13 (8–14)	0.042
White blood cell (10^3^/μL)	7 (5–9)	6 (4–9)	7 (5–8)	7 (6–11)	7 (5–9)	0.054
Neutrophil (%)	71 (61–82)	54 (49–65)	66 (58–79)	73 (62–85)	72 (61–82)	0.001
Lymphocyte (%)	20 (11–29)	37 (25–40)	25 (16–32)	19 (10–29)	19 (11–27)	<0.001
Platelet Count (10^3^/μL)	224 (170–286)	238 (209–242)	227 (176–287)	240 (175–325)	214 (166–272)	0.050
Albumin (mg/dL)	4 (3–4)	4 (3–5)	4 (4–5)	4 (3–4)	4 (3–4)	0.061
AST (IU/L)	31 (21–48)	23 (19–35)	31 (20–50)	38 (23–59)	29 (21–41)	0.053
ALT (IU/L)	25 (15–39)	25 (16–44)	22 (13–36)	27 (16–48)	25 (16–38)	0.346
Total Bilirubin (mg/dL)	1 (0–2)	<1 (0–1)	<1 (<0–1)	<1 (<0–1)	1 (0–1)	0.110
Creatinine (mg/dL)	1 (1–1)	1 (1–1)	1 (1–1)	1 (1–1)	1 (1–2)	0.054
Lactate Dehydrogenase (U/L)	278 (223–392)	125 (121–135)	278 (222–407)	360 (268–496)	259 (214–334)	<0.001
C-reactive protein (mg/L)	22 (8–56)	-	35 (9–82)	30 (8–79)	19 (7–48)	0.003
(ii) Age > 65 years						
Median (IQR)						
Hemoglobin (g/dL)	12 (7–14)	11 (7–14)	12 (9–13)	11 (8–13)	12 (8–14)	0.56
White blood cell (10^3^/μL)	7 (5–9)	6 (4–9)	6 (5–8)	7 (6–10)	7 (5–9)	0.023
Neutrophil (%)	67 (58–78)	54 (49–65)	64 (56–74)	69 (60–81)	71 (58–79)	0.018
Lymphocyte (%)	23 (14–32)	37 (25–40)	27 (19–34)	21 (13–32)	20 (12–29)	<0.001
Platelet Count (10^3^/μL)	236 (186–289)	238 (209–242)	231 (188–285)	257 (195–338)	227 (182–276)	0.006
Albumin (mg/dL)	4 (4–5)	4 (3–5)	4 (4–5)	4 (3–4)	4 (4–4)	<0.001
AST (IU/L)	27 (19–45)	23 (19–35)	25 (19–44)	38 (20–59)	26 (18–38)	0.226
ALT (IU/L)	24 (14–37)	25 (16–44)	21 (12–35)	27 (15–48)	26 (16–35)	0.346
Total Bilirubin (mg/dL)	0 (0–1)	0 (0–1)	0 (0–1)	0 (0–1)	1 (0–1)	0.015
Creatinine (mg/dL)	1 (1–1)	1 (1–1)	1 (1–1)	1 (0–1)	1 (1–1)	0.200
Lactate Dehydrogenase (U/L)	262 (213–366)	125 (125–125)	254 (205–366)	355 (255–460)	253 (199–314)	<0.001
C-reactive protein (mg/L)	16 (5–39)	-	26 (8–82)	17 (5–49)	15 (5–31)	0.011
(iii) Age > 65 years						
Median (IQR)						
Hemoglobin (g/dL)	12 (7–14)	10 (8–14)	11 (8–13)	12 (8–14)	13 (8–14)	0.16
White blood cell (10^3^/μL)	7 (5–10)	-	7 (6–10)	8 (6–11)	7 (5–9)	0.369
Neutrophil (%)	75 (64–84)	-	76 (63–89)	76 (66–89)	74 (64–83)	0.034
Lymphocyte (%)	16 (9–26)	-	17 (8–26)	16 (6–24)	17 (10–26)	0.257
Platelet Count (10^3^/μL)	206 (158–274)	-	205 (153–298)	217 (155–300)	206 (158–266)	0.669
Albumin (mg/dL)	4 (3–4)	-	4 (3–4)	3 (3–4)	4 (3–4)	0.069
AST (IU/L)	34 (24–52)	-	49 (34–62)	40 (27–64)	32 (23–45)	0.001
ALT (IU/L)	26 (16–40)	-	27 (15–38)	28 (18–48)	24 (15–39)	0.498
Total Bilirubin (mg/dL)	1 (0–1)	-	0 (0–1)	1 (0–1)	1 (0–1)	0.169
Creatinine (mg/dL)	1 (1–2)	-	1 (1–2)	1 (1–2)	1 (1–2)	0.808
Lactate Dehydrogenase (U/L)	305 (232–408)	-	365 (286–515)	396 (280–511)	264 (216–351)	<0.001
C-reactive protein (mg/L)	33 (10–64)	-	48 (9–84)	45 (20–105)	24 (9–60)	<0.001

**Table 4:** Oxygen respiratory support within 48 h of COVID-19 patients by outbreak period.

Oxygen Respiratory Support	Overall, n (%)	SARS-CoV-2 Variant, n (%)				*p*-Value
		Pre-Alpha	Alpha	Delta	Omicron	
(i) All ages						
N	596	1	131	148	316	
Canular	274 (46.0)	1 (100.0)	46 (35.1)	66 (44.5)	161 (50.9)	0.106
High Flow Nasal Canular	268 (45.0)	0 (0.0)	70 (53.5)	67 (45.3)	131 (41.5)	
Mask with bag	22 (3.7)	0 (0.0)	5 (3.8)	9 (6.1)	8 (2.5)	
ET tube	32 (5.4)	0 (0.0)	10 (7.6)	6 (4.1)	16(5.1)	
(ii) Age < 65 years						
N	222	1	74	67	80	
Canular	103 (46.4)	1 (100.0)	28 (37.8)	32 (47.8)	42 (52.5)	0.192
High Flow Nasal Canular	102 (45.9)	0 (0.0)	41 (55.4)	30 (44.7)	31 (38.8)	
Mask with bag	6 (2.7)	0 (0.0)	0 (0.0)	4 (6.0)	2 (2.5)	
ET tube	11 (5.0)	0 (0.0)	5 (6.8)	1 (1.5)	5 (6.2)	
(iii) Age > 65 years						
N	374	0	57	81	236	
Canular	171 (45.7)	0 (0.0)	18 (31.5)	34 (42.0)	119 (50.4)	0.320
High Flow Nasal Canular	166 (44.4)	0 (0.0)	29 (50.9)	37 (45.6)	100 (42.4)	
Mask with bag	16 (4.3)	0 (0.0)	5 (8.8)	5 (6.2)	6 (2.5)	
ET tube	21 (5.6)	0 (0.0)	5 (8.8)	5 (6.2)	11 (4.7)	

**Table 5:** The median and mean of length of stay (LOS) of COVID-19 patients by outbreak time period.

Length of Stay (LOS)	Overall	SARS-CoV-2 Variant				*p*-Value
		Pre-Alpha	Alpha	Delta	Omicron	
(i) All ages						
Median (IQR)						
Length of stay (days)	9 (6–12)	14 (14–14)	11 (9–14)	10 (8–14)	8 (5–10)	<0.001
Mean ± S.D.						
Length of stay (days)	10.8 ± 9.3	12.4 ± 4.2	12.3 ± 6.2	11.3 ± 6.6	10.0 ± 11.1	0.008
(ii) Age < 65 years						
Median (IQR)						
Length of stay (days)	10 (7–13)	14 (14–14)	12 (10–15)	11 (9–13)	8 (6–10)	<0.001
Mean ± S.D.						
Length of stay (days)	12.6 ± 23.1	12.8 ± 3.9	13.7 ± 8.3	16.6 ± 44.5	9.7 ± 9.3	0.038
(iii) Age > 65 years						
Median (IQR)						
Length of stay (days)	9 (6–13)	-	10 (9–14)	11 (8–16)	8 (5–11)	<0.001
Mean ± S.D.						
Length of stay (days)	11.5 ± 11.2	-	12.0 ± 6.9	12.7 ± 8.1	11.0 ± 12.6	0.376

## Data Availability

The original data generated in this study are included in this article. Further enquiries can be directed to the corresponding author.

## References

[1] Ranieri V.M., Rubenfeld G.D., Thompson B.T., Ferguson N.D., Caldwell E., Fan E., Camporota L., Slutsky A.S., ARDS Definition of Task Force (2012). Acute respiratory distress syndrome: The Berlin Definition. JAMA.

[2] Li Q., Guan X., Wu P., Wang X., Zhou L., Tong Y., Ren R., Leung K.S., Lau E.H., Wong J.Y. (2020). Early transmission dynamics in Wuhan, China, of novel Coronavirus-infected pneumonia. N. Engl. J. Med..

[3] Islam M.A. (2021). Prevalence and characteristics of fever in adult and pediatric patients with coronavirus disease 2019 (COVID-19): A systematic review and meta-analysis of 17515 patients. PLoS ONE.

[4] Saniasiaya J., Islam A. (2021). Prevalence of Olfactory Dysfunction in Coronavirus Disease 2019 (COVID-19): A Meta-analysis of 27,492 Patients. Laryngoscope.

[5] Wang B., Andraweera P., Elliott S., Mohammed H., Lassi Z., Twigger A., Borgas C., Gunasekera S., Ladhani S., Marshall H.S. (2023). Asymptomatic SARS-CoV-2 Infection by Age: A Global Systematic Review and Meta-analysis. Pediatr. Infect. Dis. J..

[6] U.S. Centers for Disease Control and Prevention (CDC) (2020). Interim Clinical Guidance for Management of Patients with Confirmed Coronavirus Disease (COVID-19).

[7] Rambaut A., Holmes E.C., O’Toole Á., Hill V., McCrone J.T., Ruis C., du Plessis L., Pybus O.G. (2020). A dynamic nomenclature proposal for SARS-CoV-2 lineages to assist genomic epidemiology. Nat. Microbiol..

[8] Center for Disease Control and Prevention (2021). SARS-CoV2 Variant Classifications and Definitions. https://stacks.cdc.gov/view/cdc/105817.

[9] hCoV-19 Variants Dashboard Data to Produce the Charts Was Updated on 18 March 2024 11: 30UTC.VOC/VOI/VUM Relative Frequencies Over Time. https://gisaid.org/hcov-19-variants-dashboard/.

[10] Government of Canada SARS-CoV-2 Variants: National Definitions, Designations and Public Health Actions. https://www.canada.ca/en/public-health/services/diseases/2019-novel-coronavirus-infection/health-professionals/testing-diagnosing-case-reporting/sars-cov-2-variants-national-definitions-classifications-public-health-actions.html.

[11] Parums V. (2021). Editorial: Revised World Health Organization (WHO) Terminology for Variants of Concern and Variants of Interest of SARS-CoV-2. Med. Sci. Monit..

[12] World Health Organization (WHO) (2022). WHO Thailand Weekly Situation Update No.224-COVID-19 Situation, Thailand. [Online]. https://cdn.who.int/media/docs/default-source/searo/thailand/2022_02_23_tha-sitrep-224-covid-19.pdf?sfvrsn=b3447f88_5.

[13] Hu F.-H., Jia Y.-J., Zhao D.-Y., Fu X.-L., Zhang W.-Q., Tang W., Hu S.-Q., Wu H., Ge M.-W., Du W. (2023). Clinical outcomes of the severe acute respiratory syndrome coronavirus 2 Omicron and Delta variant: Systematic review and meta-analysis of 33 studies covering 6 037 144 coronavirus disease 2019-positive patients. Clin. Microbiol. Infect..

[14] Miyashita K., Hozumi H., Furuhashi K., Nakatani E., Inoue Y., Yasui H., Karayama M., Suzuki Y., Fujisawa T., Enomoto N. (2023). Changes in the characteristics and outcomes of COVID-19 patients from the early pandemic to the delta variant epidemic: A nationwide population-based study. Emerg. Microbes Infect..

[15] World Health Organization (2021). COVID-19 Clinical Management: Living Guidance. https://www.google.com/url?sa=t&source=web&rct=j&opi=89978449&url=https://iris.who.int/handle/10665/338882&ved=2ahUKEwj2mP_BmcWJAxX8SWwGHa4PJ-gQFnoECBsQAQ&usg=AOvVaw27E6Q-8m3hTvpPcCRUPjAY.

[16] Sheleme T., Bekele F., Ayela T. (2020). Clinical Presentation of Patients Infected with Coronavirus Disease 19: A Systematic Review. Infect. Dis. Res. Treat..

[17] Jassat W., Karim S.S.A., Mudara C., Welch R., Ozougwu L., Groome M.J., Govender N., von Gottberg A., Wolter N., Wolmarans M. (2022). Clinical severity of COVID-19 in patients admitted to hospital during the omicron wave in South Africa: A retrospective observational study. Lancet Glob. Health.

[18] Buchan S.A., Tibebu S., Daneman N., Whelan M., Vanniyasingam T., Murti M., Brown K.A. (2022). Increased Household Secondary Attacks Rates with Variant of Concern Severe Acute Respiratory Syndrome Coronavirus 2 Index Cases. Clin. Infect. Dis..

[19] Tegally H., Wilkinson E., Giovanetti M., Iranzadeh A., Fonseca V., Giandhari J., Doolabh D., Pillay S., San E.J., Msomi N. (2021). Detection of a SARS-CoV-2 variant of concern in South Africa. Nature.

[20] Campbell F., Archer B., Laurenson-Schafer H., Jinnai Y., Konings F., Batra N., Pavlin B., Vandemaele K., Van Kerkhove M.D., Jombart T. (2021). Increased transmissibility and global spread of SARS-CoV-2 variants of concern as at June 2021. Eurosurveillance.

[21] Duong B.V., Larpruenrudee P., Fang T., Hossain S.I., Saha S.C., Gu Y., Islam M.S. (2022). Is the SARS CoV-2 Omicron Variant Deadlier and More Transmissible Than Delta Variant?. Int. J. Environ. Res. Public Health.

[22] Sadeghi F., Halaji M., Shirafkan H., Pournajaf A., Ghorbani H., Babazadeh S., Ezami N., Fallhpour K., Fakhraie F., Gorjinejad S. (2024). Characteristics, outcome, duration of hos-pitalization, and cycle threshold of patients with COVID-19 referred to four hospitals in Babol City: A multicenter retrospective observational study on the fourth, fifth, and sixth waves. BMC Infect. Dis..

[23] Department of Medical Services, Ministry of Public Health Guidelines for Clinical Practice, Diagnosis, Treatment and Prevention of Healthcare-Associated Infection in Response to Patients with COVID-19 Infection for Medical and Healthcare Personnel 2023. https://covid19.dms.go.th/backend///Content/Content_FIle/Bandner_(Big)/Attach/25660418150440PM_CPG_COVID-19_v.27_n_18042023.pdf.

[24] Shirafkan H., Sadeghi F., Halaji M., Rahmani R., Yahyapour Y. (2023). Demographics, clinical characteristics, and outcomes in hospitalized patients during six waves of COVID-19 in Northern Iran: A large cohort study. Sci. Rep..

[25] Surveillances V. (2020). The epidemiological characteristics of an outbreak of 2019 novel coronavirus diseases (COVID-19)—China, 2020. China CDC Wkly..

[26] Namdar A.B., Keikha M. (2022). BA.2.12.1 is a new omicron offshoot that is a highly contagious but not severe disease. Ann. Med. Surg..

[27] Siordia J.A. (2020). Epidemiology and clinical features of COVID-19: A review of current literature. J. Clin. Virol..

[28] Zizza A., Recchia V., Aloisi A., Guido M. (2021). Clinical features of COVID-19 and SARS epidemics. A literature review. J. Prev. Med. Hyg..

[29] Kimball A., Hatfield K.M., Arons M. (2020). Asymptomatic and presymptomatic SARS-CoV-2 infections in residents of a long-term care skilled nursing Facility King County, Washington, March 2020. MMWR Morb. Mortal. Wkly. Rep..

[30] Karuniawati A., Pasaribu A.P., Lazarus G., Irawany V., Nusantara D.U., Sinto R., Nasution M.J., Lubis M.R., Nurfitri E., Mutiara M. (2024). Characteristics and clinical outcomes of patients with pre-delta, delta and omicron SARS-CoV-2 infection in Indonesia (2020–2023): A multicentre prospective cohort study. Lancet Reg. Health-S. Asia.

[31] Di Mascio D., Khalil A., Saccone G., Rizzo G., Buca D., Liberati M., Vecchiet J., Nappi L., Scambia G., Berghella V. (2020). Outcome of coronavirus spectrum infections (SARS, MERS, COVID-19) during pregnancy: A systematic review and me-ta-analysis. Am. J. Obstet. Gynecol. MFM.

[32] Abdullah F., Myers J., Basu D., Tintinger G., Ueckermann V., Mathebula M., Ramlall R., Spoor S., De Villiers T., Van der Walt Z. (2022). Decreased severity of disease during the first global omicron variant covid-19 outbreak in a large hospital in tshwane, south africa. Int. J. Infect. Dis..

[33] Kumar D., Verma S., Mysorekar I.U. (2023). COVID-19 and pregnancy: Clinical outcomes; mechanisms, and vaccine efficacy. Transl. Res..

[34] Deng J., Ma Y., Liu Q., Du M., Liu M., Liu J. (2022). Association of Infection with Different SARS-CoV-2 Variants during Pregnancy with Maternal and Perinatal Outcomes: A Systematic Review and Meta-Analysis. Int. J. Environ. Res. Public Health.

[35] Villar J., Conti C.P.S., Gunier R.B., Ariff S., Craik R., Cavoretto P.I., Rauch S., Gandino S., Nieto R., Winsey A. (2023). Pregnancy outcomes and vaccine effectiveness during the period of omicron as the variant of concern, INTERCOVID-2022: A multinational, observational study. Lancet.

[36] Laird-Gion J., Dionne A., Gauvreau K., Baker A., Day-Lewis M., de Ferranti S., Friedman K., Khan N., Mahanta S., Son M.B. (2023). MIS-C across three SARS-CoV-2 variants: Changes in COVID-19 testing and clinical characteristics in a cohort of U.S. children. Eur. J. Pediatr..

[37] Department of Disease Control, Ministry of Public Health Thailand Situation Update Coronavirus COVID-19 in API (JSON/CSV Data Format). https://covid19.ddc.moph.go.th/.

[38] COVID-19 Vaccine. MOPH Immunization Center. Thailand. Division of General Communicable Diseases. https://ddc.moph.go.th/uploads/ckeditor2//files/Daily%20report%202021-07-31.pdf.

